# Key community eye health messages: Viral infections of the eye

**Published:** 2020-03-30

**Figure:**
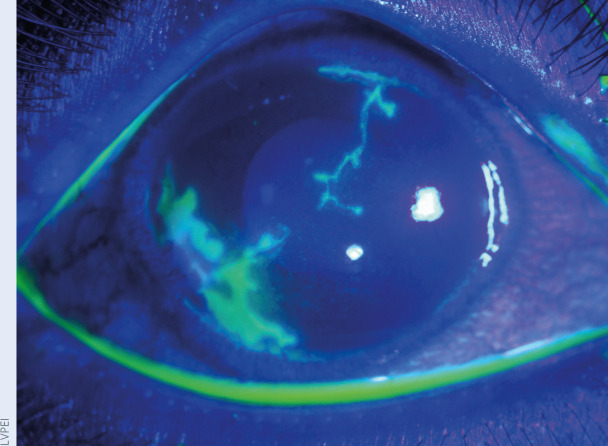


Herpes simplex keratitis is common. It can present in different forms. Antiviral treatment is recommended. Do **not** use steroids in dendritic or geographic ulcers.

**Figure:**
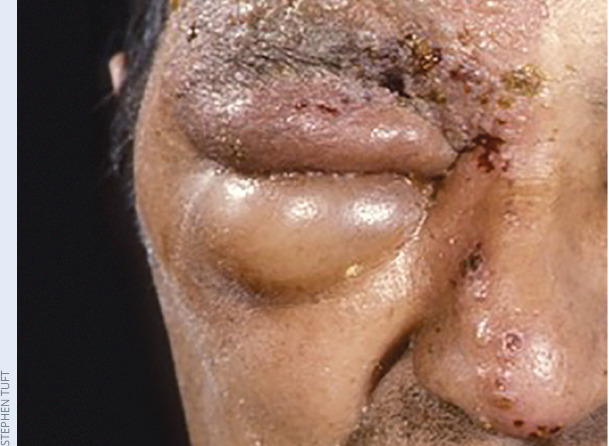


Herpes zoster ophthalmicus can cause corneal damage and iritis, especially when the nasociliary nerve is affected as shown by a rash on the tip of the nose.

**Figure:**
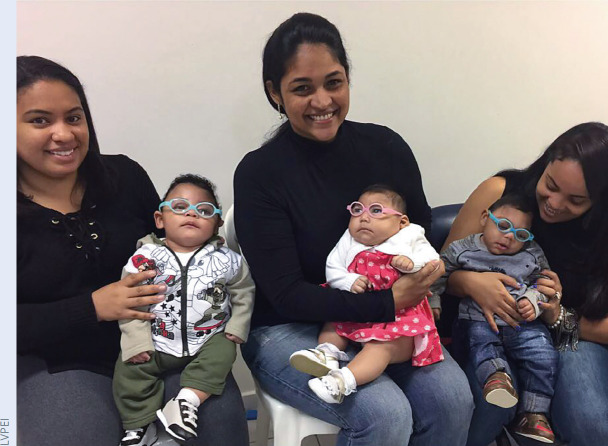


Zika infection in pregnancy can cause congenital Zika syndrome in a newborn baby. All babies suspected of having been exposed to Zika virus in utero should have a full eye examination.

